# A Microfluidic Long-Period Fiber Grating Sensor Platform for Chloride Ion Concentration Measurement

**DOI:** 10.3390/s110908550

**Published:** 2011-09-02

**Authors:** Jian-Neng Wang

**Affiliations:** Department of Construction Engineering, National Yunlin University of Science and Technology, Douliou 64002, Taiwan; E-Mail: wangjn@yuntech.edu.tw; Tel.: +886-5-5342601 ext. 4723; Fax: +886-5-5312049

**Keywords:** microfluidic, long-period fiber grating (LPFG), chloride ion, transmitted optical power, random walk coefficient, bias stability, 07.60.Vg, 42.81.-I, 47.27.nf

## Abstract

Optical fiber sensors based on waveguide technology are promising and attractive in chemical, biotechnological, agronomy, and civil engineering applications. A microfluidic system equipped with a long-period fiber grating (LPFG) capable of measuring chloride ion concentrations of several sample materials is presented. The LPFG-based microfluidic platform was shown to be effective in sensing very small quantities of samples and its transmitted light signal could easily be used as a measurand. The investigated sample materials included reverse osmosis (RO) water, tap water, dilute aqueous sample of sea sand soaked in RO water, aqueous sample of sea sand soaked in RO water, dilute seawater, and seawater. By employing additionally a chloride ion-selective electrode sensor for the calibration of chloride-ion concentration, a useful correlation (R^2^ = 0.975) was found between the separately-measured chloride concentration and the light intensity transmitted through the LPFG at a wavelength of 1,550 nm. Experimental results show that the sensitivity of the LPFG sensor by light intensity interrogation was determined to be 5.0 × 10^−6^ mW/mg/L for chloride ion concentrations below 2,400 mg/L. The results obtained from the analysis of data variations in time-series measurements for all sample materials show that standard deviations of output power were relatively small and found in the range of 7.413 × 10^−5^−2.769 × 10^−3^ mW. In addition, a fairly small coefficients of variations were also obtained, which were in the range of 0.03%–1.29% and decreased with the decrease of chloride ion concentrations of sample materials. Moreover, the analysis of stability performance of the LPFG sensor indicated that the random walk coefficient decreased with the increase of the chloride ion concentration, illustrating that measurement stability using the microfluidic platform was capable of measuring transmitted optical power with accuracy in the range of −0.8569 mW/ $\sqrt{h}$ to −0.5169 mW/ $\sqrt{h}$. Furthermore, the bias stability was determined to be in the range of less than 6.134 × 10^−8^ mW/h with 600 s time cluster to less than 1.412 × 10^−6^ mW/h with 600 s time cluster. Thus, the proposed LPFG-based microfluidic platform has the potential for civil, chemical, biological, and biochemical sensing with aqueous solutions. The compact (3.5 × 4.2 cm), low-cost, real-time, small-volume (∼70 μL), low-noise, and high-sensitive chloride ion sensing system reported here could hopefully benefit the development and applications in the field of chemical, biotechnical, soil and geotechnical, and civil engineering.

## 1. Introduction

In recent years, the technology of fiber optic sensors has been applied to the field of structural monitoring, infrastructure assessment, and some industrialized sectors [1–4]. The detection of chloride ion is of great interest in a variety of industrial applications, including civil and environmental monitoring, soil and geotechnical engineering, plant and crop science, food, bio-based and industrial chemistry. In particular, the salt damage to concrete structures due to the corrosion of reinforced bars in the marine environment is normally caused by the penetration of chloride ion into the concrete. Industrial health monitoring of chloride-ion distributions along different locations inside concrete structures in harsh saltwater environment is essential for the maintenance and management of reinforced concrete structures [5,6]. Traditional methods of chloride-ion measurement involve titrations, colorimeters, fluorescence quenching, or ion-selective electrodes. Most of the available sensors are based on an electrochemical reaction that has low sensitivity and has difficult in sensing ions at low concentration levels. Since chloride remains an important ion for sensing applications in physiological samples, much effort have been involved to develop suitable chloride ion sensors for use in chemical sensor applications. However, as has happened with other types of ion-concentration optical sensors [7–11], only a limited number of highly selective optical chloride sensors have been reported [6,12–16]. The most commonly reported techniques, in conjunction with an optical fiber, are based on the development of sensitive sensing materials as matrix for entrapment of optical transducers that can provide the function of selective exaction and chemical recognition of the analyte, a few examples such as fluorescence quenching of a fluorophore immobilized on a polymeric matix [8], Na^+^ ions adsorption on sol-gen porous film [11], and ion-selective membranes deposited on dielectric waveguides [9,10]. Among them, ionophore-based sensors are the most attractive and are routinely used for the analysis of aqueous samples. However, the number of anion-selective ionophores that have useful selectivity is still quite limited. For example, so far only a few number of selective ionophores for chloride exist, such as indium(III) metalloporphyrins [17], alkyltin(IV) derivatives [18], a hydrogen-bonding ionophore [19], and a [14] mercuracarborand-3 (MC-3) ionophore [12,13]. Chloride-ion concentrations have been reported using the mercuracarborand ionophore with mechanistic insights into the development of an optical chloride sensor [13]. A chloride optical sensor based on a membrane with an optical response to chloride has been developed and the membrane contains two luminophores displaying two largely different decay times [14]. The use of chloride selective compounds associated with an optical sensor to detect chloride ion concentration has been studied [15]. A novel transduction chemistry for preparing optical anion-selective polymeric films that respond reversibly and selectively to chloride ion activity was demonstrated [16]. In addition, a wireless, passive, remote-query sensor for monitoring sodium hypochlorite (bleach, NaOCl) solutions has been reported [20]. The sensor is comprised of a magnetically-soft ferromagnetic ribbon, coated with a layer of polyurethane and alumina, having a large and nonlinear permeability that supports higher-order harmonics in response to a time varying magnetic field. This sensor platform could enable long-term monitoring of sodium hypochlorite concentrations in the environment [20].

However, most of the reported methods require substantial or complicated instrumentation which in general involves tedious laboratory procedures and cumbersome chemical processes. In recent years, considerable research attention has focused on long-period fiber gratings (LPFGs) for many applications due to their low insertion losses, low back-reflection, polarization independence, and relatively simple fabrication. The LPFG is extremely sensitive to the refractive index (RI) of the material surrounding the cladding surface, thus allowing it to be used as ambient RI or chemical solution sensors [6,21–23]. The advantages of this type of grating sensor are their simple fabrication and easy interrogation. Moreover, combined with the advantages of optical fibers, the sensor has the potential capability for on-site, real-time, and remote sensing without the need for handling the substance under test, can be easily multiplexed to enable high-throughput screening of chemical reactions, and has the potential use for disposable sensors.

Many types of microfluidic chips with different sensing mechanisms have been used to detect the concentration of chemical species such as chloride ion, salt, and sucrose. A micro-electro-mechanical systems (MEMS)-based microfluidic system for measuring chemical concentration, fluid density and specific gravity has been developed to measure methanol/water concentration *versus* density at 20 °C [24]. The realization of a liquid tunable long-period grating using multiphase droplet microfluidics that is integrated onto a microfluidic chip to test de-ionized (DI) water and CaCl_2_ solutions using a 1,550 nm light source is reported in [25].

This study addresses the development of an LPFG-based microfluidic chip for chloride ion measurement using different kinds of water and chloride ion concentration solutions. The use of hybrid microfluidic chips and LPFG sensors are shown to have the advantages of sensing very small quantities of samples, detections with high resolution and sensitivity, low cost, short times for data acquisition and analysis—only transmitted optical power, and simple experimental setup devices for different sample materials with or without chloride ion concentration. Furthermore, the combination of a multi-D-shaped optical fiber and a microfluidic chip has successfully demonstrated the feasibility of fabricating a class of high sensitive refractive-index sensor using only light intensity—transmitted optical power [26]. In this scheme, only a laser diode source associated with an optical power meter is required for signal interrogation. Expensive and high precision apparatus such as a broadband amplified spontaneous emission (ASE) fiber source associated with a high-resolution optical spectrum analyzer (OSA) is not necessary. Thus, the advantages of the microfluidic platform are simplicity, compactness, low cost, small volume, high integration, and time savings for experiments. In this paper, we focused on the use of LPFG-based microfluidic chip as chloride ion concentration sensor and the light intensity of the LPFG in the output power was used to quantify the chloride ion concentrations of different kinds of waters, aqueous samples of sea sand soaked in RO water, and seawater. The wavelength shift information was not used to quantify the chloride-ion concentration or the refractive index of sample materials. Additionally, a chloride ion-selective electrode (ISE) sensor (CL-BTA, Vernier) was employed and calibrated with high (1,000 mg/L) and low (10 mg/L) standard solutions. The chloride ISE has a resolution about 0.7% of reading (1.8 ± 0.013 or 35,500 ± 250 mg/L) and was used to measure chloride-ion concentrations in various samples with sea sand or seawater. Results from these two sensors show a useful correlation (R^2^ = 0.975) between the ISE-measured chloride concentration and LPFG-measured transmitted light intensity at a wavelength of 1,550 nm (*vide infra*). Thus, to the best if our knowledge, a microfluidic platform with a long-period fiber grating was able to successfully perform for the first time chloride ion concentration measurements using different waters, aqueous samples of sea sand soaked in RO water, and seawater.

## 2. Microfluidic Platform

### 2.1. Long-Period Fiber Grating

In general, an LPFG usually has a periodic refractive index modulation along the core of a single-mode fiber, with a typical perturbation of Δ*n* ∼ 10^−4^, periods between 100 μm^−1^ mm and length of 2–4 cm. The LPFG couples light from a guided fundamental core mode (LP*_01_*) to different forward-propagating cladding modes (HE*_1m_*) in an optical fiber, which is given by phase-matching condition [23]:

(1) $$\beta_{\mathrm{core}}^{01}-\beta_{\mathrm{cladding}}^{1m}=\frac{2\pi }{\Lambda }\;\;\;\;m=2,3,4,....$$

where $\beta_{\mathrm{core}}^{01}$ and $\beta_{\mathrm{cladding}}^{1m}$ are propagating constants of the fundamental core mode and *m*th cladding mode, respectively, and Λ is the period of grating. The coupling of the light into the cladding region generates a series of resonant bands centered at wavelength *λ_m_* in the transmission spectrum. The center wavelengths *λ_m_* of an attenuation band are solutions of the following equation [23]:

(2) $$\lambda_{m}=\left[{\bar{n}}_{\mathrm{core}}^{01}\left(n_{1},n_{2},\lambda_{m}\right)-{\vec{n}}_{\mathrm{cladding}}^{1m}\left(n_{2},n_{s},\lambda_{m}\right)\right]\Lambda$$

where ${\bar{n}}_{\mathrm{core}}^{01}\left(n_{1},n_{s},\lambda_{m}\right)$ is the effective index of the fundamental core mode at the wavelength of *λ_m_*, which is also dependent on the core refractive index *n*_1_ and cladding refractive index *n_2_*. In Equation (2) ${\vec{n}}_{\mathrm{cladding}}^{1m}\left(n_{2},n_{s},\lambda_{m}\right)$ is the effective refractive index of the *m*th cladding mode at the wavelength *λ*_m_, which is related to cladding refractive index *n*_2_ and the refractive index of the surrounding medium *n_s_*. When the concentration or the refractive index of the surrounding medium changes, also ${\vec{n}}_{\mathrm{cladding}}^{1m}\left(n_{2},n_{s},\lambda_{m}\right)$ changes and a shift in the central wavelength can be obtained. The cladding modes are very sensitive to change in the refractive index of the ambient (surrounding) environment.

Since Vengsarkar *et al.* [27] first reported the fabrication and some characteristics of LPFGs in 1996, various techniques for inscription of LPFGs in different types of optical fibers have been demonstrated [28–34], just to name a few examples such as ultraviolet (UV) laser exposure [28], Nd-YAG laser grooving [29], CO_2_ laser irradiation [30], femtosecond laser exposure [31], electric arc discharge [32], ion beam implantation [33], and mechanically induced method [34]. The LPFGs are typically characterized by their period, resonance wavelength, resonance width (full-width at half maximum or FWHM) and strength (peak amplitude at resonance wavelength). The achieved resonance width and strength for the fabricated LPFGs is shown Table 1. In this study, the LPFGs were fabricated by the electric-arc discharge method [22] with hydrogen-free Corning SMF-28 fibers. LPFGs are especially suitable for measurements and applications when liquids or solutions undergo a change in temperature or RI which can be used for sensing temperatures, liquid-levels, and chloride ions [4,6,21–23,35–40]. The experimental setup for LPFGs fabrication consists of a computer-controlled electric-arc discharge associated with a translation stage, a broadband ASE fiber source and a high-resolution OSA (ANDO AQ6315A) used to monitor *in situ* the transmission loss as the grating was written.

**Figure 1.**
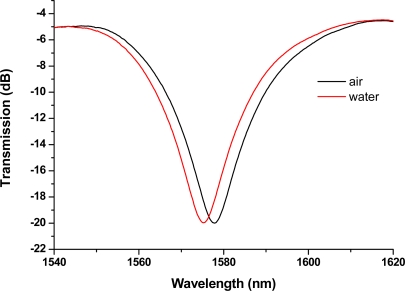
Transmission spectra of an LPFG measuring sample materials in air or in water. The resonance wavelength of the LPFG in air = 1,577.8 nm.

The electric-arc discharge-induced LPFGs were about 2.2–3.6 cm long and their grating periods were about 600 μm. The transmission spectrum was interrogated during the writing and its characteristics such as insertion loss, resonance peak wavelength, and peak depth were analyzed after the grating was written. With suitable fabrication parameters such as electric-arc discharge current, discharge time, grating period, and scan speed, the resulting resonance wavelengths ranging from 1,200 nm to 1,600 nm with a greater than about 20 dB peak depth were obtained. The cladding mode profiles of LPFGs are very sensitive to changes in the refractive index of the ambient (surrounding) environment. Figure 1 shows the transmission spectra of an LPFG measuring sample materials in air or in water at 25 Celsius. The resonance wavelength of the LPFG in air is 1,577.8 nm. This LPFGs sensor was used to conduct our light intensity—transmitted optical power measurements for all experimental sample materials presented in this paper.

### 2.2. Microfluidic Chip

Microfluidics is the science and technology of systems that process or manipulate small (about nanoliter or picoliter volume) amounts of fluids, using channels with dimensions of tens to hundreds of micrometers. The advantages of microfluidic technology include the ability to use very small quantities of samples and reagents, and to carry out separations and detections with high resolution and sensitivity, low cost, short times for analysis, and small footprints for the analytical devices [41–43]. The D-shaped fiber-optic sensing system with a microfluidic chip has successfully been used to perform the RI sensing measurements for sucrose solutions with refractive indices in the range of 1.333 to 1.403 [26].

Recently, Rindorf *et al.* [44] first presented the integration of a photonic crystal fiber (PCF)-based optical sensor with a lab-on-chip component, where a 16-mm-long piece of microstructured optical fiber (MOF) is incorporated into an optic-fluidic coupler chip, allowing the continuous control of liquid flow through the MOF and simultaneous optical characterization. This biochip, with a capacity of analyzing sample volumes down to 300 nL, was successfully used in a DNA capture experiment [44]. In addition, Rindorf *et al.* [45] demonstrated that a long-period grating in a photonic crystal fiber (PCF-LPG) can be used for sensitive biochemical sensing in the detection of biomolecules, in which a much smaller sample volume is possible by using the holes in the cladding as microfluidic channels. Using the device, the thicknesses of a monolayer of poly-l-lysine and double-stranded DNA was measured and reported. The PCF-LPG grating has shown a sensitivity of approximately 1.4 nm/nm in terms of the shift in resonance wavelength in nm per nm thickness of biomolecule layer [45]. Furthermore, Rindorf and Bang [46] reported a highly sensitive refractometer based on a long-period grating in a large-mode-area PCF. This sensor shows a maximum sensitivity of 1,500 nm/refractive index unit at a refractive index of 1.33 and a minimal detectable index change of 2 × 10^−5^. The high sensitivity is obtained by infiltrating the sample into the holes of the PCF to give a strong interaction between the sample and the probing field. The CO_2_-induced LPFGs are damage gratings [45,46]. Rindorf and Bang [47] investigated the sensitivity of photonic crystal fiber grating sensors for biosensing, refractive index, strain, and temperature sensing, in which it is found that the dispersion plays a central role in determining the sensitivity, and that dispersion may enhance or suppress sensitivity as well as change the sign of the resonant wavelength shifts. A quality factor, Q, rather than the sensitivity, has been proposed for characterizing the performance of long period gratings sensors [47]. The Q factor is defined as the ratio of resonant wavelength shift to the FWHM of resonance band. It is shown that for PCFs infiltrated with samples, the *Q* for refractive index sensing and biosensing may be improved by several orders of magnitude by an appropriate choice of the hole diameter and pitch.

**Figure 2.**
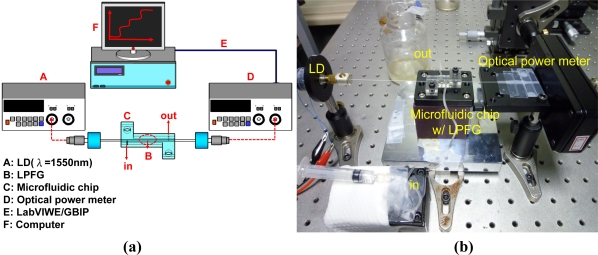

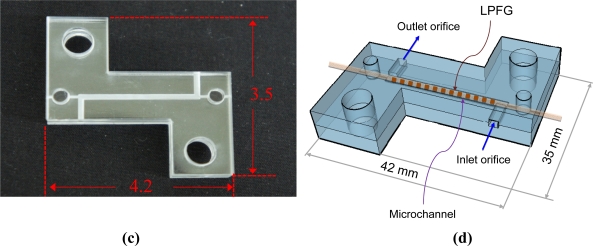
**(a)** Schematic of the experimental setup for chloride ion measurements using an LPFG-based microfluidic chip system; **(b)** Photograph of the LPFG-based microfluidic system; **(c)** Photograph of the microfluidic chip; **(d)** 3D illustration of the structure and fluidic operation of microfluidic chip (material: PMMA).

A microfluidic system with an LPFG, as a chemical sensor, has been established to carry out chloride ion measurements. Figure 2(a) shows the schematic of the experimental setup for sample material measurements with an LPFG-based microfluidic chip system and Figure 2(b) is a photograph of this system, where the microfluidic chip is made of poly(methyl methacrylate) (PMMA). Figure 2(c) is a photograph of the microfluidic chip and Figure 2(d) illustrates the structure and fluidic operation of the microfluidic chip, which comprises of a pair of S-shape plates about 42 mm long and 35 mm wide, fabricated by a computer-controlled engraving machine. The two S-shaped plates with a microchannel made on one of the S-shape plates were then bonded together by hot-pressing technology, applying heat in conjunction with mechanical pressure in an oven over 150 °C for several minutes. The inlet and outlet orifices of the microfluidic chip are used to infuse the solution with analyte (if any) flowing through the LPFG sensing fiber which is placed at the center of the reaction microchannel. The reaction microchannel was 200 μm high and 200 μm wide with a length of 28 mm. When integrated with a grooved (D-type or U-type) optical fiber modified with self-assembled gold nanoparticles which are functionalized with a receptor, this type of microfluidic chip has been shown, both numerically and experimentally, to be an optimum design as fiber-optic localized plasmon resonance biosensors to reduce sample and reagent volume, to shorten response time and analysis time, and to increase sensitivity [43]. Therefore, the microfluidic platform formed with the integration of the LPFG with a microfluidic chip could provide efficient performance on biochemical binding and could induce chaotic advection to enhance the micro-fluid mixing in the microchannel. This compact and low-cost sensor has potential applications in medical diagnostics, biochemical sensing, and environmental monitoring, and can hopefully benefit the development and application of different fields, such as material testing, plant and crop science, and civil and geotechnical engineering.

## 3. Materials and Experiments

### 3.1. Materials

The LPFG-based microfluidic system was used to measure the attenuated light intensity in output power or transmission loss of different chloride ion concentration aqueous samples from sea sand and seawater, which were sampled at Mailiao Township of Yunlin County, Taiwan. Seawater contains about 34,000 ppm of dissolved salts. The chlorides of sodium, magnesium, and calcium, about 90.3%, are the most abundant dissolved salts [5]. Thus, the study of those experimental materials with sea sand and seawater presented here was mainly aimed to the real life application in civil engineering such as reinforced concrete structures immersed in seawater or soils and aggregates containing chloride ions. It is not intended for bio-sensing field which normally requires highly selective and sensitive methods to sense target ions at low concentration.

The sample numbers (No.) and their corresponding material were as follows: No. 1: air, No. 2: 50-mL RO water, No. 3: 50-mL tap water, No. 4: 50-mL dilute aqueous sample of sea sand soaked in RO water, sampling from 1,000-g oven-dry sea sand immersed in 1,600-mL RO water; No. 5: 50-mL aqueous sample of sea sand soaked in RO water, sampling from 1,000-g oven-dry sea sand immersed in 800-ml RO water, No. 6: 50-mL dilute seawater, sampling from 50-mL seawater mixed with 50-mL RO water, and No. 7: 50-mL seawater. Since the higher sample Nos. possessed much higher chloride ion concentrations, the time-series experiment was conducted in the order of Nos. 1 to 7.

**Figure 3.**
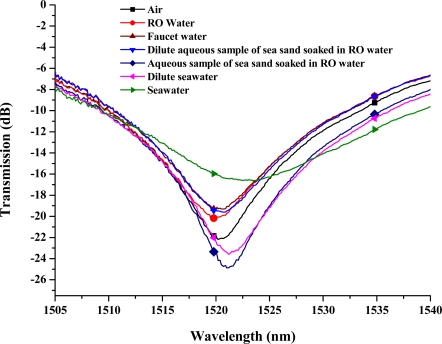
Transmission spectra of an LPFG-based microfluidic chip measuring sample materials with or without chloride ions. The resonance wavelength of the LPFG in air is 1,520.5 nm.

As reported previously [26], the combination of a multi-D-shaped optical fiber and a microfluidic chip has successfully demonstrated the feasibility of fabricating a class of high sensitive refractive-index sensor using only light intensity—transmission loss [26]. The advantages of our current microfluidic platform presented here are simplicity, compactness, low cost, real time measurements and small volume (∼70 μL), without using a broadband ASE fiber source and a high-resolution OSA, and time savings for experiments. Thus, we aimed at the use of the LPFG-based microfluidic chip as a low-cost and effective chloride ion concentration sensor based on light intensity modulation. Though LPFG wavelength shifts have been shown to be a reliable, repeatable, and accurate measurand for several different field applications [22], for simplicity the wavelength shifts were not used to measure the chloride ion values of different sample materials. Nevertheless, Figure 3 illustrates the feasibility of using wavelength shifts of an LPFG-based microfluidic chip to measure different sample materials with or without chloride ions.

The small size (3.5 × 4.2 cm) and highly integrated microfluidic platform was designed to perform the chloride ion concentration measurements—transmitted optical power with different kinds of waters, and aqueous samples from sea sand and seawater. Thus, this experiment only needs an optical power meter with a broadband laser diode (LD) light source at a wavelength of 1,550 ± 30 nm. The testing was done in the following order: sample Nos. 1–3 (without chloride ion) were measured for 30 min, and then sample Nos. 4–7 (with chloride ions) for 60 min, respectively. Thus, the chloride ion measurements of sample materials were in time series as the sequence of Nos. 1 to 7. Prior to the testing for sample Nos. 4–7, we used RO water to flush the microchannel and keep it clean. That could ensure the sample materials were under stable conditions. For each run of optical power measurement, there were a series of 1,800–3,600 data points.

### 3.2. Experimental Setup

**Figure 4.**
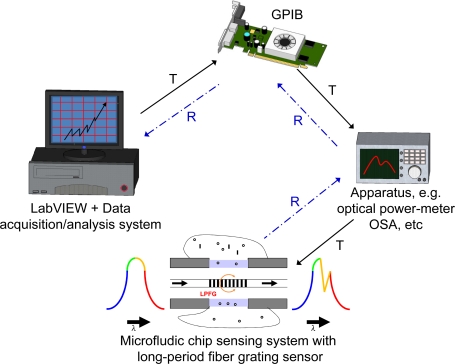
Schematic of an automatic LPFG-based sensing or monitoring system using the microfluidic platform (T: transmitting; R: receiving).

The experimental setup for chloride ion measurements with the LPFG-based microfluidic system consists of an LPFG, a microfluidic chip, an optical power meter (Advantest Q8221) with a broadband LD light source, a computer using the LabVIEW software, and a GPIB controller connected with the optical power meter [see Figure 2(a)]. Figure 4 shows the schematic of an automatic LPFG-based sensing or monitoring system using this microfluidic platform, where T and R represent transmitting and receiving processes, respectively.

For the samples we studied, apart from the target anions such as chloride ions any other impurities will modify the refractive index and appear as a change in the transmittance, each with its own particular calibration to the concentration of that given impurity. In our samples associated with seawater or sea sand about 90% or more of chemical compositions are chlorides of sodium, magnesium, and calcium. To overcome this issue, a chloride ISE sensor (CL-BTA, Vernier), in addition to the LPFG sensor, was employed to calibrate the chloride-ion concentration in reverse osmosis water and aqueous solutions of sea sand or seawater.

The results obtained by the two sensors (*vide infra*), showing a strong linear relationship (R^2^ = 0.975) between transmitted light intensity and chloride ion concentration, suggest that the other compositions or impurities in the samples either have the similar responses as those of chlorides or have small or negligible contributions. In the former case, to precisely extract the calibration parameters and exclude the contributions from any impurities it may require to establish one further correlation between the ISE-measured chloride concentration and LPFG-measured light intensity transmitted through the LPFG using different chloride ion-containing aqueous solutions such as NaCl (aq), MgCl_2_ (aq), and CaCl_2_ (aq). An experiment to resolve this issue will be conducted shortly and results will be reported elsewhere.

During the course of data measurement we have maintained the strain, bending, and temperature conditions quite stable in the laboratory so that they were not of concern in this work. For precise measurement, we kept the experimental setup and sample materials at a constant ambient temperature (within 0.1 °C fluctuation). The LPFG sensing fiber was placed inside the microfluidic chip and a small fixed magnitude of tension was applied to minimize bending of the fiber. Therefore, the results reported here were not influenced by the effects of temperature, strain and bending [see Figure 2(b)].

### 3.3. Statistics and Random Walk Coefficient

Several traditional statistic methods have been implemented to characterize the variations in those time-series transmitted power measurements, including average (mW), standard deviation (mW), range (mW), and coefficient of variation (CV, %). In addition, random walk coefficient (RWC, mW/ $\sqrt{h}$) and bias stability (BS, mW/h with time cluster), which are well suited for analysis of stability performance [48–50], were employed to evaluate the long-term variations in concentration measurement. RWC is defined as the slope of Allan variance plot (Allan deviations of transmitted optical power versus time cluster) before Allan deviation approaches the minimum value. The Allan variance is computed for many different sampling times ranging from 1 s to 600 s or more. The bias stability is then obtained as the minimum Allan deviation and occurs at the corresponding time cluster [48–50]. These two parameters, RWC and BS, were used to analyze the chloride ion measurement stability—attenuated light intensity in output power of the microfluidic platform for sample Nos. 1–7 and were compared with the results from traditional statistics, such as average (mW), standard deviation (mW), range (mW), and co efficient of variation (CV, %).

## 4. Results and Discussion

**Figure 5.**
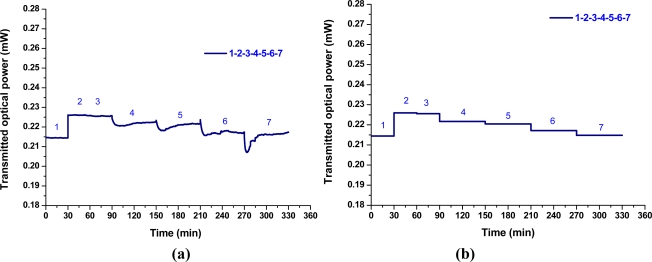
**(a)** Plot of transmitted optical power *versus* time for measurements of sample materials; **(b)** Plot of average transmitted optical power *versus* time for measurements of sample materials.

**Figure 6.**
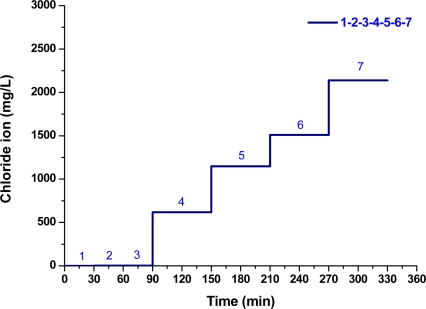
Plot of average chloride ion concentration *versus* time for measurements of sample materials.

**Figure 7.**
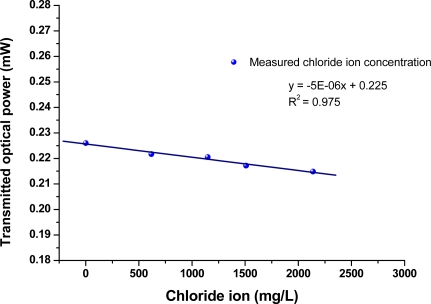
Plot of chloride ion concentration *versus* transmitted optical power (sample No. 2: RO water and Nos. 4–7: aqueous samples associated with sea sand and seawater).

In this study, an LPFG sensor of resonance wavelength at 1,577.8 nm were used to collect data from the time series of transmitted power measurement for Nos. 1–7, which responded linearly and reversibly with respect to the change of chloride ion concentration. The results of chloride ion measurements were plotted and analyzed for the transmitted optical power and chloride ion concentration. Figure 5(a) shows the time-series plot of transmitted optical power (mW) versus time (min) for measurements of sample materials, in which the sample No. is marked with blue font. The plot of average optical power *versus* time for sample materials is shown in Figure 5(b). In addition, the results obtained using the chloride ISE sensor have been plotted as the average chloride ion concentrations *versus* time for different solutions (see Figure 6). Figure 6 shows the graph of chloride concentration (by mg/L, ppm, or and percent concentration by weight) obtained by the chloride ISE *versus* time (min). With a linear regression analysis, the graph of chloride ion concentrations versus transmitted optical power is shown in Figure 7. The results from these two sensors show that the relationship between transmitted light intensities in output power and different chloride ion concentration solutions was close to linear (R^2^ = 0.975). The transmitted optical power of the sample solutions decreased as the chloride ion concentration increased. Thus, the proposed LPFG-based microfluidic platform possessed the potential to measure the different chloride ion concentrations of an aqueous solution.

**Figure 8.**
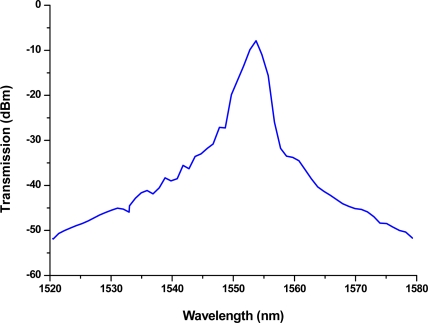
The spectrum of laser diode light source with a wavelength centered at 1550 ± 30 nm.

Note that, as shown in Figure 8, the laser diode used for measurement was a broadband light source with a wavelength centered at 1,550 ± 30 nm, which covers a wide range of wavelengths and has sufficient intensity at a wavelength in the tail of the attenuation band (1,577.8 nm) shown in Figure 1 for measurements.

Using this wavelength associated with the microfluidic LPFG sensor, we have found a relatively linear relationship between the transmitted light intensity and chloride ion concentration in the samples. The is due to the fact that the cladding modes are very sensitive to changes in the ambient refractive index (ARI) of the surrounding environment, particularly when ARI is higher than that of the cladding. It has been shown theoretically and experimentally that the change of resonant wavelength or sensitivity monotonically increases as the ARI approaches that of the cladding [23,51–53]. In our study, as shown in Figures 1 and 3, we found that amplitude (or intensity) change of resonant wavelength follows the similar trend as that of wavelength change. Thus, compared with the data shown in Figures 1 and 3 from the two different LPFGs, the one with the intensity at resonance wavelength of 1,577.8 nm was a better sensor for chloride measurements.

**Figure 9.**
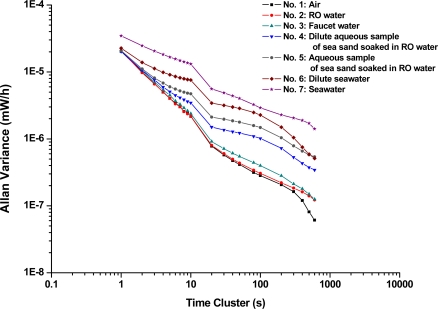
Plot of Allan variance versus time cluster for measurements of sample materials.

Figure 9 displays the RWCs for measurements of sample Nos. 1–7. All RWCs (mW/ $\sqrt{h}$), BSs (mW/h), and the average values (mW), standard deviations (mW), ranges (mW), and CVs (%) of sample Nos. 1–7 were summarized in Table 2. The data variations were relatively small. The standard deviations for sample Nos. 1–7 were in the range of 7.413 × 10^−5^−2.769 × 10^−3^ mW. The normalized statistics—coefficient of variations (%) for sample Nos. 1–7 were in the range of 0.03%–1.29% and the CV value decreased as the chloride ion concentration of sample materials decreased.

The results from stability tests of transmitted optical power show that the random walk coefficients of power variations were in the range of −0.8569 mW/ $\sqrt{h}$ to −0.5169 mW/ $\sqrt{h}$ for sample Nos. 1–7. The RWCs of sample Nos. 1–7, which are the slopes of Figure 9, decreased as the chloride ion concentrations increased. Thus, the trend displayed that the chloride ion concentration increased as the RWC decreased (or conversely, as CV increased). It can be seen that the bias stability was in the range of less than 6.134 × 10^−8^ mW/h with 600 s time cluster to less than 1.412 × 10^−6^ mW/h with 600 s time cluster for sample Nos. 1–7. Results presented here illustrate that the measurement stability of sample Nos. 1–7 using the microfluidic platform, was capable of measuring transmitted optical power with accuracy in the range of −0.8569 mW/ $\sqrt{h}$ to −0.5169 mW/ $\sqrt{h}$. The bias stability of measuring transmitted optical power were found in the range of less than 6.134 × 10^−8^ mW/h with 600 s time cluster to less than 1.412 × 10^−6^ mW/h with 600 s time cluster.

## 5. Conclusions

A microfluidic system for chloride ion measurement using a long-period fiber grating is presented. The LPFG, as a high sensitive ion concentration or refractive-index sensor to detect changes in the surrounding RI, was fabricated with an electric-arc discharge system. Several sample materials were used for this study which included reverse osmosis water, tap water, dilute aqueous sample of sea sand soaked in RO water, aqueous sample of sea sand soaked in RO water, dilute seawater, and seawater. The realization of the sensor is through the measurement of transmitted light intensity of the sensing fiber. A chloride ISE sensor was employed and calibrated with high (1,000 mg/L) and low (10 mg/L) standard solutions to measure chloride-ion concentrations in osmosis water, dilute aqueous sample of sea sand soaked in RO water, aqueous sample of sea sand soaked in RO water, dilute seawater, and seawater. When exposing the microfluidic LPFG sensor to sample solutions of increasing chloride ion concentration, the sensor response increases linearly (R^2^ = 0.975). A fairly useful correlation is established between the ISE-measured chloride ion concentration and the LPFG-measured light intensity. The sensitivity of the LPFG fiber sensor by transmitted power interrogation was determined to be 5.0 × 10^−6^ mW/mg/L. Results obtained from this LPFG-based microfluidic platform illustrate the feasibility of effective chloride ion detection in very small (∼70 μL) seawater samples, in which the majority of chemical components (∼90%) are chlorides. For the study of the stability performance of chloride ion measurement, random walk coefficients and bias stabilities were implemented and proposed to compare with traditional statistics, which include averages, standard deviations, ranges, and coefficient of variations. The coefficient of variations were found in the range of 0.03%–1.29% and decreased with the decrease of the chloride ion concentration of sample materials. The stability parameter RWC were calculated to be in the range of −0.8569/ $\sqrt{h}$ to −0.5169 mW/ $\sqrt{h}$ and decreased with the increase in chloride ion concentration.

Such microfluidic electric arc-induced LPFG fiber sensor exhibit some promising features for sensor applications, such as simple, small size (3.5 × 4.2 cm), real time, small volume (∼70 μL), low noise (∼7.413 × 10^−5^−2.769 × 10^−3^ mW), excellent measurement stability (−0.8569 mW/ $\sqrt{h}$ to −0.5169 mW/ $\sqrt{h}$) and bias stability (less than 6.134 × 10^−8^ mW/h to less than 1.412 × 10^−6^ mW/h with 600 s time cluster). Therefore, studies presented here successfully demonstrate the feasibility of fabricating a class of high sensitive chloride-ion sensor based on the LPFG-based microfluidic platform written by electric arc discharge, which has the potential for chemical, biological, and biochemical sensing with aqueous solutions. The compact, highly integrated, low cost, small volume, and high sensitive chloride ion sensing system reported here could hopefully benefit the development and applications in the field of chemical, biotechnical, soil and geotechnical, and civil engineering.

Further improvements will be made by fabricating the microfluidic system as a highly selective chloride sensor suited for measuring a wider range of samples. Note that it is not a trivial work for the proposed LPFG system, simply relying on the performance of a microfluidic LPFG sensor alone, to limit the response strictly to the presence of chlorides. When a wider range of samples are considered, the LFPG sensor can further be fabricated as a highly selective chloride sensor, similar to those biosensor-based methods, by binding (or immobilizing) a thin layer of chloride-sensing material (e.g., nanoparticles, quantum dots, or optical anion-selective polymeric film/membrane) on the cladding surface of the LPFG fiber that respond reversibly and selectively to chloride ion activity. To further fabricate the LFPG sensor as a highly selective chloride sensor is encouraging and will be studied shortly.

## Figures and Tables

**Table 1.** Review of the quality of reported LPFGs.

**Method**	**Period (μm)**	**Resonance width (nm)**	**Strength width (dB)**
UV laser [28]	400	51.5	−18
Nd-YAG laser [29]	275	44.3	−36
CO_2_ laser [30]	450	20.9	−8
fs laser [31]	460	42.9	−12
Electric discharge [32]	680	45.8	−11
Ion implantation [33]	170	76.9	−15
mechanically induced [34]	630	48.4	−13

**Table 2.** Summary of statistics and random walk coefficients for measurements of sample materials.

		**Statistics and random walk coefficients**				
**(No.): Sample (material)**	**Average (mW)**	**Standard Deviation (mW)**	**Range^1^ (mW)**	**CV^2^ (%)**	**Random walk coefficient^3^ mW/**$\sqrt{h}$	**Bias stability^4^ (mW/h; with the corresponding time cluster)**
(1): Air	2.145 × 10^−1^	7.413 × 10^−5^	3.458 × 10^−4^	3.456 × 10^−2^	−0.8569	<6.134 × 10^−8^; 600 s
(2): RO water	2.260 × 10^−1^	1.042 × 10^−4^	5.203 × 10^−4^	4.612 × 10^−2^	−0.7876	<1.222 × 10^−7^; 600 s
(3): Tap water	2.256 × 10^−1^	1.133 × 10^−4^	5.197 × 10^−4^	5.024 × 10^−2^	−0.7808	<1.253 × 10^−7^; 600 s
(4): Dilute aqueous sample of sea sand soaked in RO water	2.217 × 10^−1^	7.344 × 10^−4^	3.893 × 10^−3^	3.312 × 10^−1^	−0.5889	<3.432 × 10^−7^; 600 s
(5): Aqueous sample of sea sand soaked in RO water	2.205 × 10^−1^	1.202 × 10^−3^	5.235 × 10^−3^	5.453 × 10^−1^	−0.5277	<5.408 × 10^−7^; 600 s
(6): Dilute seawater	2.172 × 10^−1^	9.869 × 10^−4^	8.042 × 10^−3^	4.544 × 10^−1^	−0.5563	<5.108 × 10^−7^; 600 s
(7): Seawater	2.148 × 10^−1^	2.769 × 10^−3^	1.026 × 10^−2^	1.289	−0.5169	<1.412 × 10^−6^; 600 s

1 Range = The difference between maximum and minimum transmitted power values;

2 CV = Standard deviation of transmitted power values/average of transmitted power values: coefficient of variation;

3 Random walk coefficient (RWC) is defined as the slope of Allan variance plot (Allan deviations of transmitted power versus time clusters) before Allan deviation approaches the minimum value;

4 Bias stability (BS) is then obtained as the minimum Allan deviation and occurs at the corresponding time cluster and BS is typically described within the minimum Allan deviation with the corresponding time cluster. These two parameters were used to evaluate the sensing performance of microfluidic platform for all sample materials.
